# Efficacy of in-person versus digital enhanced lifestyle interventions in adults with overweight and obesity

**DOI:** 10.1016/j.obpill.2024.100133

**Published:** 2024-10-16

**Authors:** Diego Anazco, Maria A. Espinosa, Lizeth Cifuentes, Blake Kassmeyer, Tara M. Schmidt, Sima Fansa, Alejandro Campos, Elif Tama, William S. Harmsen, Maria D. Hurtado, Donald D. Hensrud, Andres Acosta

**Affiliations:** a Precision Medicine for Obesity Program, Division of Gastroenterology and Hepatology, Department of Medicine, Mayo Clinic, Rochester, MN, USA; b Quantitative Health Sciences, Mayo Clinic, Rochester, MN, USA; c Healthy Living Program, Integrative Medicine and Health Mayo Clinic, Rochester, MN, USA; d Division of Endocrinology, Diabetes, Metabolism, and Nutrition, Department of Medicine Mayo Clinic, Jacksonville, FL, USA; e Division of General Internal Medicine Mayo Clinic, Rochester, MN, USA

**Keywords:** Obesity, Lifestyle interventions, Mobile health, Electronic health

## Abstract

**Background:**

Lifestyle interventions (LIs) are the cornerstone for obesity management. The Mayo Clinic Diet (MCD) offers two approaches for LIs: the In-Person LI (IPLI) and the Digital Enhanced LI (DELI). The IPLI includes a 2-day in-person program with monthly follow-ups, whereas the DELI provides on-demand digital tools. The comparative efficacy of these approaches is currently unknown.

**Methods:**

This retrospective study included two cohorts of adults with a body mass index (BMI) of ≥25 kg/m^2^ and weight metrics at least 3 months after starting either the IPLI or DELI program. The primary endpoint was the total body weight loss percentage (TBWL%) at 6 months.

**Results:**

The study included 133 participants in the IPLI cohort (mean age 46.3 years, 65.4 % female, BMI 36.4) and 9603 in the DELI cohort (mean age 60.1 years, 85.0 % female, BMI 33.1). The DELI group achieved superior TBWL% at 1, 3, and 6 months compared to the IPLI group (3.4 % vs. 1.5 %, 4.7 % vs. 2.4 %, 5.3 % vs. 2.9 %, respectively; p < 0.001). After adjusting for age, gender, and starting weight, the DELI group maintained a higher TBWL% (difference 2.0 %; 95 % CI [1.0, 3.0], p < 0.001) and a greater proportion of participants achieved >5 % TBWL at 6 months (OR 1.66; 95 % CI [1.08, 2.55], p < 0.023).

**Conclusion:**

The DELI approach resulted in superior weight loss outcomes compared to the IPLI. Further research is needed to explore how digital tools can improve weight loss effectiveness.

## Graphical abstract

**Figure:**
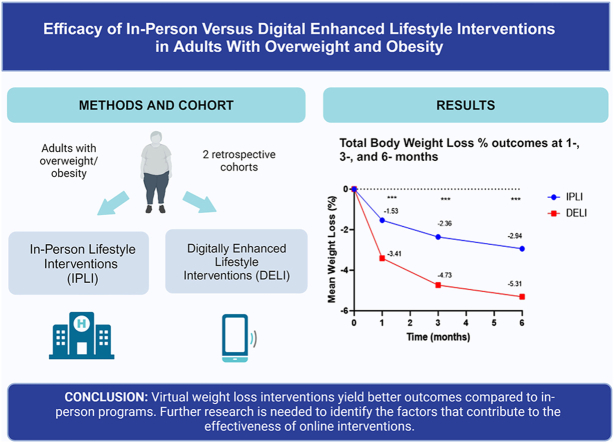

## 1 Introduction

Obesity is a chronic and heterogeneous disease that has continued to escalate to become a worldwide epidemic causing a substantial healthcare and financial burden. It has been estimated that over one billion people in the world live with obesity, with significant increases in the prevalence of obesity seen across most countries over the last three decades [1]. Projections show an alarming upward tendency, estimating that by 2030, obesity will affect almost half of U.S. adults [2]. In the U.S. alone, obesity-related healthcare costs were estimated at $480 billion annually, reflecting a significant obstacle towards a sustainable healthcare system [3].

Lifestyle interventions (LIs) are the cornerstone for obesity management [4]. LIs have been shown to be effective in the mid-to long-term prevention and treatment of obesity leading to a significant reduction in body weight and cardiovascular risk factors [5,6]. However, there is considerable variability in the response to LIs for the management of obesity, and many patients fail to achieve or maintain clinically significant weight loss [7,8]. Current guidelines for weight management recommend a calorie deficit of 500kcal–750 kcal per day based on predicted energy requirements [5]. However, given the variability in response to LIs, the Academy of Nutrition and Dietetics has recently recommended that medical nutrition therapy should be individualized to optimize outcomes [9].

While in-person lifestyle interventions have shown effectiveness when addressing obesity and associated comorbidities [6,10], the advent of digital platforms and eHealth initiatives offer promising opportunities to reach and engage participants at a broader scale, while decreasing cost when compared to traditional in-person interventions [11,12]. There is evidence that web-based interventions can be used effectively to treat overweight and obesity and are comparable to in-person interventions [[13], [14], [15], [16]]. However, digital interventions for weight management are highly variable [14], and as a result, further research assessing the efficacy of comparable in-person vs. digital LIs is needed. The Mayo Clinic Diet encompasses a comprehensive LI program available as either an in-person or a virtual approach. Both interventions share the same framework, consisting of an individualized and structured program with two distinct phases: “Lose It”, designed to induce sufficient weight loss during the active phase; and “Live It”, to facilitate the development of effective lifestyle modifications to prevent weight regain. The Mayo Clinic Diet In-Person Lifestyle Intervention (IPLI) is delivered by a multi-disciplinary group of health professionals over two-days at the Mayo Clinic in Rochester, Minnesota. On the other hand, the Mayo Clinic Diet Digital-Enhanced Lifestyle Intervention (DELI) provides on-demand education and digital resources through an online platform, which comprises self-monitoring tracking tools, group coaching sessions, notifications, emails, and forums to help with adherence. This retrospective study aims to evaluate the weight loss effectiveness of a DELI compared to a structured IPLI. We hypothesized that the interactive web-based program would result in comparable weight loss outcomes as compared to the in-person intervention.

## 2 Material and methods

### 2.1 Study design and population

We conducted a retrospective cohort study examining two groups that underwent either an IPLI or a DELI following the Mayo Clinic Diet framework. For the IPLI group, we searched the electronic medical records to collect the demographic, anthropometric, and weight loss outcomes of adults with overweight and obesity who underwent a weight reduction phase at home and completed the 2-day in-person training at the Mayo Clinic Healthy Living Program in Rochester, Minnesota, between January 2014 to December 2021. Medical examinations were conducted at baseline and every four weeks for three months, in which patients' anthropometric data was registered. The Mayo Clinic Institutional Research Review Board (IRB) approved the study. Informed consent was waived due to minimal risk for participants.

For the DELI group, we gathered demographic and anthropometric characteristics, usage data, and self-reported questionnaires from the Mayo Clinic Diet Online Platform. Upon enrollment for the virtual program, participants agreed to use their information in research. We collected information from participants who began the DELI program between 01/01/2022 and 10/23/2022 and had initial weight data and weight values after at least three months. Patients' anthropometric information was self-reported at registration, and participants were encouraged to constantly record their weight in the online application.

**Fig. 1:**
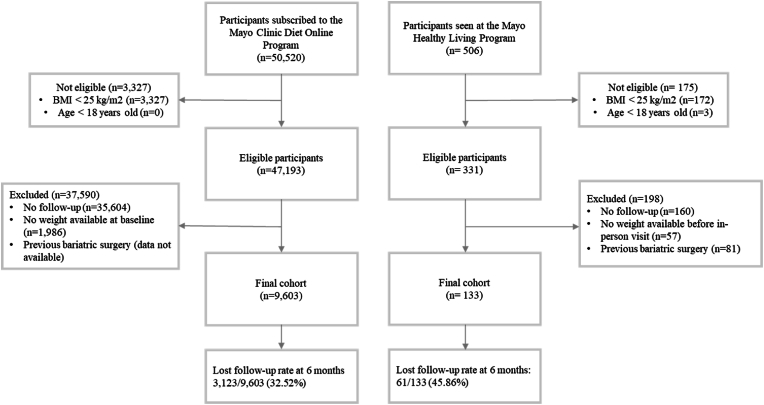
Participant flowchart.

We included adult participants with a body mass index (BMI) ≥25 kg/m^2^ that participated in either weight loss program and had weight data at least three months after program initiation. For the in-person modality, we excluded all patients that had history of bariatric surgery, were using antiobesity medications, had an endoscopic or surgical weight loss revisional procedure, or denied authorization for use of their medical records in research (Fig. 1). For the DELI cohort, we did not have information regarding previous history of bariatric surgery, endoscopic or surgical weight loss revisional procedures, or concomitant use of antiobesity medications (see Fig. 1).

### 2.2 Interventions

#### 2.2.1 In-person lifestyle intervention (IPLI)

Participants completed the “Lose It” phase at home to promote weight loss through dietary adjustments, increased activity, and resilience-related topics. After that, they completed a 2-day face-to-face lifestyle intervention at the Healthy Living Program in Rochester, Minnesota, which covered a sequence of tasks that participants are instructed to follow during the “Live-it” phase. This intensive IPLI consisted of a multi-disciplinary and individualized health and wellness program with three essential pillars: medical nutrition therapy, physical activity, and resilience-related behavioral interventions. This program includes a physical activity assessment, individualized health and wellness coaching, and several didactic sessions covering themes such as healthy living practices, significance of physical activity and exercise, and resiliency and self-efficacy. Participants meet with a dietitian to establish a diet plan and program goals; and with a physical therapist to conduct a strength and balance assessment to provide an activity plan at home. Moreover, participants attend weekly visits with a wellness coach for 12 weeks to discuss participant's strengths and motivation for change, challenges, and personal goals.

The follow-up phase to maintain weight loss involved changing eating habits and behaviors based on decreasing portion size (except vegetables and fruits), reducing overall calorie intake, and increasing food quality, with a recommendation to consume daily fresh or frozen vegetables and fruits. Participants were instructed to use a weight pyramid to guide their food choices and consume a specific amount of servings from various food group in the pyramid to plan their meals. The physical activity recommendation was to increase the daily activity, increase movement along the day, and increase exercise time to at least 30, and preferably 60 minutes on most days of the week.

#### 2.2.2 Digital enhanced lifestyle intervention (DELI)

Participants self-enrolled in the online weight-loss program based on the principles of the Mayo Clinic Diet. The user registration process collects information on age, sex, weight loss goals, and reasons for weight loss.

Users were instructed to go through 2 distinct phases: a 2-week “Lose-It” phase, where they were encouraged to change 15 habits to promote rapid weight loss in a safe and healthy manner. This phase was followed by an ongoing “Live-It” phase in which people turn these habits into long-term lifestyle changes with additional guidelines. The diet is structured around three meals (breakfast, lunch, and dinner) and snacks each day using a food group system, whereby portions of food are presented as standard servings for each food group. Individuals are assigned to 1 of 4 calorie goals depending on their starting weight (1200, 1400, 1600, or 1800 kCal/day). There are six food groups: fruits, vegetables, protein/dairy, carbohydrates, fats, and sweets. The program encourages unlimited healthy, low-energy, dense fruit, and vegetable consumption. Other food groups have an upper limit of servings/day.

Ongoing support is available from program features, including written nutrition content, weekly emails, regular webinars, recipes for the meal plans, a habit tracker for the Lose It phase, food tracker with keyword search and barcode functionality, activity tracker linked with a device, weight tracker with the ability to link with a wireless weight scale, and support from a private 10.13039/100005801Facebook group.

### 2.3 Data collection

For the IPLI cohort, data was manually abstracted from the electronic medical records through a retrospective chart review. Weight was collected at the time of the first visit, after 1-month (±7 days), after 3-months (±15 days), and after 6-months (±45 days). We collected data from the Mayo Clinic Diet Online Platform for the DELI cohort. Users provided informed consent to participate in the research during initial sign-up. Participants were enrolled based on self-interest in weight loss. Baseline height, weight, and demographics were self-reported during the registration process. Participants self-reported their weight in the mobile application. Information was gathered at baseline, after 1-month, (±7 days), after 3-months (±15 days), and after 6-months (±45 days).

### 2.4 Study endpoints

The study's primary endpoint was the total body weight loss percentage (TBWL%) at 6 months of follow-up for patients participating in either the IPLI or the DELI program. The outcome was calculated using the following formula:

$$TBWL\%=\frac{Body\;weight\;at\;each\;time\;point-Baseline\;body\;weight\;}{Baseline\;body\;weight}\times 100$$

Basal Metabolic Rate (BMR) was calculated using the Harris-Benedict equation. Secondary endpoints included the TBWL% at 1- and 3- months, and the proportion of patients who had a reduction from the baseline body weight of ≥5 % and ≥10 % at 1-,3- and 6- months when comparing both interventions.

### 2.5 Statistical Analysis

We present continuous variables as mean and standard deviation and categorical variables as counts and proportions. For the primary endpoint, we used 10 multiple imputation datasets for missing data using the mice package in R. Additional estimates were calculated adjusting for age, gender and starting weight. Models were constructed for each of the 10 multiple imputed datasets and were combined using Rubin's rules. Unadjusted proportion estimates of patients above 5 % and 10 % TBWL were assessed using proportion tests. Linear models were constructed to assess %TBWL and logistic regression models were used for assessing weight loss above 5 % and 10 % in models adjusted for age, gender and starting weight. We reported odds ratios from these logistic regression models to measure how the odds of achieving significant weight loss changed with the predictors, while controlling for confounders. Based on parameter estimates (PE), we summarized the results with 95 % confidence intervals (95 % CI) and significance values. Significant tests were 2-sided with an α value set at .05. All analyses were performed in R 4.2.2 (R Foundation Vienna Austria). This study followed the Strengthening the Reporting of Observational Studies in Epidemiology (STROBE) reporting guideline (Supplementary Material).

## 3 Results

Baseline characteristics are included in Table 1. Of the individuals selected at baseline across both cohorts, 47,524 out of 51,026 (93.1 %) met the starting criteria for either the IPLI or DELI programs (Fig. 1). Of these 47,524 individuals, 9736 (20.5 %) met all the study criteria, which included having a baseline weight measurement and weight measurements for at least 3 months after program initiation and not meeting any exclusion criteria. The DELI cohort had 9603 out of 47,193 (20.3 %) participants meeting all the study criteria, while the IPLI cohort had 133 out of 331 (40.2 %) participants meeting these criteria. Notably, the IPLI participants had a higher average baseline BMI (36.4 [SD 6.5] kg/m^2^) compared to those in the DELI group (33.1 ± 5.9 kg/m^2^). Additionally, individuals in the DELI program were older (60.1 ± 13.1 years) than those in the IPLI program (46.3 ± 13.8 years; p < 00.001), with a greater proportion of females enrolled (85.4 % [8161/9603]) compared to the IPLI program (65.4 % [87/133]; p < 00.001). Furthermore, participants in the DELI program had a lower Basal Metabolic Rate (1624.3 ± 225.7 kcal/day) than participants in the IPLI program (2022.3 ± 429.6 kcal/day; p < 00.001). This could be related to the DELI group having a lower BMI at baseline than the IPLI group. The lost to follow-up rate at 6 months was lower for the DELI group [3123/9,603, (32.52 %)] compared to the IPLI group [61/133, (45.86 %)]. No other demographic characteristics differed between groups (Table 1).

**Table 1:** Baseline demographic and clinical information.

	**In-Person**	**Virtual**	p-value
	**N= 133**	**N= 9603**	
Age (years)	46.3 ± 13.8	60.1 ± 13.1	< 0.001^a^
Female	87 (65.4 %)	8161 (85.0 %)	< 0.001^b^
Height (cm)	171 ± 1	166 ± 1	< 0.001^a^
Race, White	121 (92.4 %)	8264 (90.3 %)	< 0.001^b^
Starting Weight (kg)	106.0 ± 21.1	91.8 ± 18.8	< 0.001^a^
Starting BMI (kg/m^2^)	36.4 ± 6.5	33.1 ± 5.9	< 0.001^a^
Obesity Class			< 0.001^b^
• Obesity Class 1	46 (34.6 %)	3229 (33.6 %)	
• Obesity Class 2	33 (24.8 %)	1758 (18.3 %)	
• Obesity Class 3	36 (27.1 %)	1188 (12.4 %)	
• Overweight	18 (13.5 %)	3428 (35.7 %)	
Basal Metabolic Rate	2022.3 ± 429.6	1624.3 ± 228.7	< 0.001^a^
Type 2 Diabetes	11 (8.3 %)	651 (6.8 %)	0.498^b^

a Two sample *t*-Test.

b Pearson's Chi-squared Test.

### 3.1 Total body weight loss

**Fig. 2:**
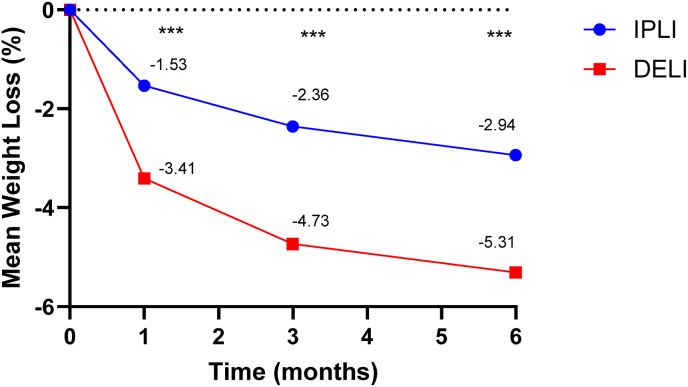
TBWL% outcomes at 1-, 3-, and 6- months. ∗∗∗: p ​< ​0.001 TBWL%: Total Body Weight Loss Percentage. IPLI: In-Person Lifestyle Intervention. DELI: Digitally Enhanced Lifestyle Intervention.

**Fig. 3:**
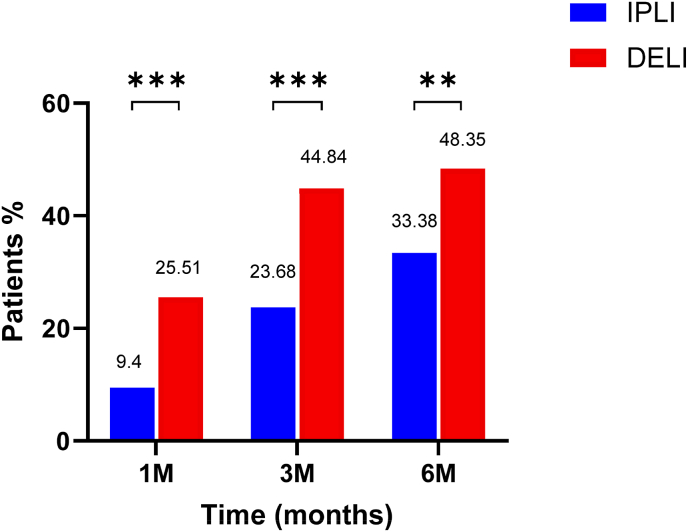
Categorical weight loss >5 % ∗∗: p ​< ​0.01; ∗∗∗: p ​< ​0.001.

**Fig. 4:**
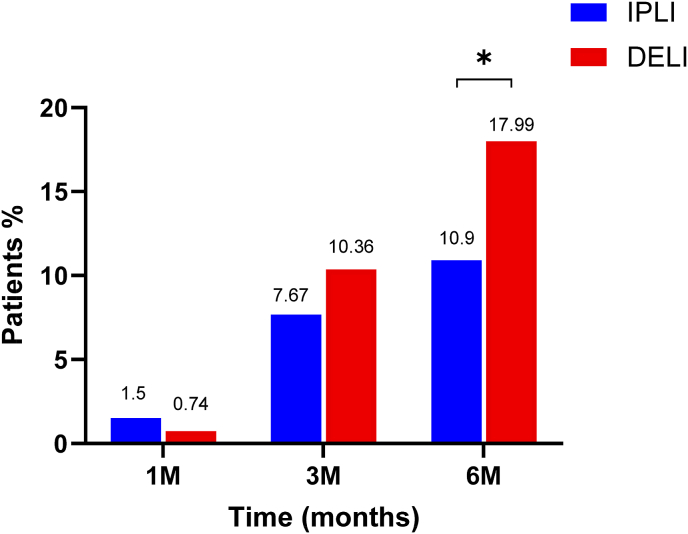
Categorical weight loss >10 % ∗: p ​< ​0.05.

The DELI group showed superior TBWL% in comparison to the IPLI group (3.4 % vs. 1.5 %, 4.7 % vs. 2.4 %, and 5.3 % vs. 2.9 %) at 1-, 3-, and 6- months, respectively (p < 0.001; see Fig. 2). At 1-, 3- and 6- months, the DELI group had a larger proportion of participants achieving a TBWL%≥ 5 % (48.4 % vs. 33.4 %, p = 0.002; see Fig. 3). At 6 -months [n = 72 (67.5 %) vs. 5480 (44.9 %)], the DELI group had a higher proportion of participants achieving a TBWL%≥ 10 % (18.0 % vs. 10.1 %, p = 0.015; see Fig. 4).

The superior TBWL% in the DELI group compared to the IPLI group remained after adjusting for age and gender (Diff = 1.99 % TBWL; 95 % CI = [1.0, 3.0]; p-value <0.001). Similarly, a higher proportion of DELI participants reached 5 % TBWL (OR = 1.66; 95 % CI [1.08, 2.55]; p-value = 0.023). A numerically higher proportion of DELI participants achieved a 10 % TBWL (OR = 1.75; 95 % CI [0.97, 3.15]; p-value = 0.06) at 6 months after adjusting for age and gender.

## 4 Discussion

In this retrospective study, we compared the weight loss outcomes of two different lifestyle interventions: digitally enhanced lifestyle interventions (DELI) and in-person lifestyle interventions (IPLI). Both lifestyle interventions were derived from the same framework incorporating the principles of The Mayo Clinic Diet. However, participants in the DELI group achieved superior weight loss, and more participants reached clinically significant weight loss milestones as compared to the IPLI group. In addition, the IPLI group presented with demographic characteristics often associated with greater weight loss success: a higher proportion of male participants [17,18] and higher baseline BMIs [19,20]. However, despite these favorable demographics, in our study, the IPLI group demonstrated inferior weight loss outcomes compared to the DELI group. These findings suggest that virtual interventions might provide specific features that could influence weight loss success.

A recent systematic review suggested that web-based interventions for weight loss are often more effective than minimal treatments [14]. The in-person lifestyle intervention (IPLI) group exhibited a slightly higher dropout rate at six months [61/133, (45.86 %)] than the digitally enhanced lifestyle intervention (DELI) group [3123/9,603, (32.52)], which may indicate improved adherence in the DELI program strategies directed towards engagement. For instance, previous studies have shown that habits such as self-weighing have been associated with superior weight loss outcomes [21]. Frequent weight reporting has been associated with greater weight loss and weight loss maintenance [[22], [23], [24], [25], [26]]. In this setting, the accessibility provided by online platforms with multiple resources to improve engagement and self-efficacy is advantageous.

The transition from a pro-obesogenic to a healthy lifestyle is the cornerstone of obesity management. Traditional face-to-face lifestyle interventions are the standard of care, and a systematic review revealed that they often lead to a mean reduction of 2.4 kg greater as compared to no or minimal interventions [27]. Also, moderate-to high-intensity interventions often produce 5 %–10 % total body weight loss. Several studies have shown that with behavioral interventions, participants achieved maximal weight loss between 6 and 12 months [5,27,28]. Real-world data with in-person lifestyle interventions resulted in a mean weight loss similar to results achieved in the IPLI group with 2.32 kg (95 % CI −2.92 to −1.72) [29]. However, in-person face-to-face interventions could be lengthy and require specialized professionals, making it challenging to support all patients, especially in a continuously expanding target population. Employing digital interventions, independently or in combination with face-to-face interactions, offers a potential solution to this limitation.

Web-based weight management interventions are a rising field of research and clinical care [30,31]. Mobile health (mHealth) refers to the use of mobile and wireless communication technologies to improve healthcare delivery [32]. Previous studies have assessed the efficacy of mHealth interventions for the management of obesity and other chronic conditions [33,34]. Numerous studies have examined the impact of web-based and face-to-face interventions separately. For instance, systematic reviews on web-based weight-loss interventions have found them effective compared to a non-technology active or no intervention control group [35,36]. The weight loss outcomes for the DELI group in our study (TBWL of 5.3 % at 6 months) are slightly superior to the outcomes previously reported in a retrospective study that evaluated the effectiveness of a structured digital 12-month program based on acceptance and commitment therapy (mean TBWL of 3.1 % at 6-months) [22]. A meta-analysis including 15 randomized control trials on personalized eHealth intervention demonstrated a superior mean weight loss of 2.8 kg (R 2.0–3.5kg) in the eHealth intervention group compared to controls (standard care or no intervention). However, a prior meta-analysis indicated a mean weight loss of 0.77kg (95 % CI −2.16 to 0.62), suggesting lesser weight reduction than in the DELI group of our study [35].

The rapid growth of mobile technology has led to mixed results regarding their effectiveness in weight loss management. Additionally, the high volume of available applications makes it difficult to evaluate these technologies thoroughly [37]. Digital interventions can differ in design and structure, which can also largely influence results, making it challenging to assess outcomes across studies. In addition, some digital interventions do not include a personalized or tailored approach. Interactive applications have shown better results than noninteractive programs. A randomized controlled trial found that an interactive web-based program was successful in achieving weight loss and enhancing body composition among adults with overweight and obesity, in contrast to a noninteractive web-based program [38]. More research is needed to compare web-based interventions to in-person interventions. This comparison could identify specific characteristics of either approach linked to weight loss success. Our study compared these two modalities to determine which could produce better weight loss outcomes.

## 5 Limitations

This study's strengths include adequate sample size and a follow-up duration of 6-months. Moreover, previous studies have usually compared in-person or web-based interventions to control groups receiving no or minimal intervention. In our study, we compared two comparable lifestyle interventions with distinct delivery methods but based on the same framework. Our study also has some limitations. Web-based programs are accessible to a broader population. Therefore, the number of participants in the DELI group significantly outnumbered those in the IPLI group. Second, missing a control group (no intervention) is a significant limitation in assessing both cohorts' effectiveness. Third, having a larger proportion of White female patients limits the generalizability of the results. Fourth, there is susceptibility to inaccurate documentation in the extraction phase due to the observational and retrospective design of this study. Fifth, excluding patients with less than 3 months follow-up might lead to an overestimation of the weight loss outcomes. Sixth, weight measurements collected in the DELI group may not be as accurate as office visits in the IPLI group. Finally, the loss to follow-up seen in this study could have impacted the ability to fully assess mid-term outcomes. In addition, more extended follow-up periods are needed to assess for weight loss maintenance. Further studies should also investigate which factors of the DELI are associated with prediction of response to the intervention.

## 6 Conclusion

The development of digital-based applications can potentially increase the accessibility to weight loss interventions while addressing multiple barriers to care. At the system level, incorporating digitally enhanced programs can help decrease the overall cost of health care without compromising outcomes. However, more studies are needed to better understand the specific characteristics of digital-based interventions that might lead to superior weight loss outcomes.

• Digitally enhanced lifestyle interventions are associated with superior weight loss outcomes and higher achievement rates of weight loss clinical milestones compared to in-person lifestyle interventions.
• Understanding the advantages of digital tools in obesity management can facilitate a better integration into clinical practice. This integration has the potential to enhance accessibility to weight loss interventions and reduce overall healthcare costs while maintaining effective outcomes.
• More studies into the specific characteristics behind these findings are needed to understand the features of digital-based interventions that could enhance weight loss outcomes, leading to more effective and personalized approaches for achieving and maintaining weight loss.

## Source of funding

10.13039/100000871Mayo Clinic Internal Funds.

## Declaration of use of artificial intelligence

During the preparation of this work the author(s) did not use generative artificial intelligence (AI) or AI-assisted technologies.

## Data availability statement

The investigators will share deidentified data that underlies the results reported in this article after deidentification upon request by bona fide researchers who provide a methodologically appropriate proposal. Proposals should be directed to acosta.andres@mayo.edu. To gain access, data requestors will need to sign a data access agreement.

## Author contributions

Diego Anazco: Conceptualization (equal), Data curation (lead). Formal Analysis (equal), Investigation (equal), Methodology (equal), Visualization (equal), Writing – Original Draft Preparation (lead), Writing – Review and Editing (equal). Maria A. Espinosa: Data curation (equal), Visualization (equal), Writing – Original Draft Preparation (equal), Writing – Review and Editing (equal). Diego Anazco and Maria A. Espinosa contributed equally to the manuscript. Lizeth Cifuentes, Sima Fansa, Wissam Ghusn, Alejandro Campos, Elif Tama, Tara M Schmidt: Data curation (equal), Writing – Review and Editing (equal). Blake Kassmeyer, William S. Harmsen: Statistical Analysis (lead), Writing – Review and Editing (equal). Donald D. Hensrud, Maria D. Hurtado, Andres Acosta: Writing – Review and Editing (equal), Supervision (lead), Visualization (lead), Project administration (lead), Investigation (lead).

## Data availability statement

The investigators will share deidentified data that underlies the results reported in this article after deidentification upon request by bona fide researchers who provide a methodologically appropriate proposal. Proposals should be directed to acosta.andres@mayo.edu. To gain access, data requestors will need to sign a data access agreement.

## Declaration of competing interest

Dr. Acosta is supported by NIH (NIH K23-DK114460). Dr Andres Acosta, and Mayo Clinic hold equity in Phenomix Sciences Inc. and are inventors of intellectual property licensed to Phenomix Sciences Inc. Dr Andres Acosta served as a consultant for Rhythm Pharmaceuticals, General Mills, Amgen, Bausch Health, RareStone; has contracts with Vivus Inc, Satiogen Pharmaceutical, and Rhythm Pharmaceutical.
