# Supplementary material for: Efficacy of in-person versus digital enhanced lifestyle interventions in adults with overweight and obesity
Source: Obes Pillars. 2024 Oct 16;12:100133. doi: 10.1016/j.obpill.2024.100133 (PMC11532308; doi:10.1016/j.obpill.2024.100133)
Supplement: Multimedia component 1 [file mmc1.pdf]

STROBE Statement—checklist of items that should be included in reports of observational studies

	Item No.	Recommendation	Page No.	Relevant text from manuscript
**Title and abstract**	1	(*a*) Indicate the study's design with a commonly used term in the title or the abstract	3	This retrospective study included two cohorts of adults
		(*b*) Provide in the abstract an informative and balanced summary of what was done and what was found	3	
**Introduction**				
Background/rationale	2	Explain the scientific background and rationale for the investigation being reported	5	
Objectives	3	State specific objectives, including any prespecified hypotheses	5	We hypothesized that the interactive web-based program would result in comparable weight loss outcomes as compared to the in-person intervention.
**Methods**				
Study design	4	Present key elements of study design early in the paper	5	We conducted a retrospective cohort study examining two groups that underwent either an IPLI or a DELI following the Mayo Clinic Diet framework.
Setting	5	Describe the setting, locations, and relevant dates, including periods of recruitment, exposure, follow-up, and data collection	5-6	For the IPLI group, we searched the electronic medical records to collect the demographic, anthropometric, and weight loss outcomes of

				adults with overweight and obesity who underwent a weight reduction phase at home and completed the 2-day in-person training at the Mayo Clinic Healthy Living Program in Rochester, Minnesota, between January 2014 to December 2021.<br>For the DELI group, we gathered demographic and anthropometric characteristics, usage data, and self-reported questionnaires from the Mayo Clinic Diet Online Platform. Upon enrollment for the virtual program, participants agreed to use their information in research. We collected information from participants who began the DELI program between 01/01/2022 and 10/23/2022 and had initial weight data and weight values after at least three months.
Participants	6	(*a*) *Cohort study*—Give the eligibility criteria, and the sources and methods of selection of participants. Describe methods of follow-up	6	We included adult participants with a body mass index (BMI)

		Case-control study—Give the eligibility criteria, and the sources and methods of case ascertainment and control selection. Give the rationale for the choice of cases and controls<br>*Cross-sectional study*—Give the eligibility criteria, and the sources and methods of selection of participants		≥25 kg/m2 that participated in either weight loss program and had weight data at least three months after program initiation. For the in-person modality, we excluded all patients that had history of bariatric surgery, were using antiobesity medications, had an endoscopic or surgical weight loss revisional procedure, or denied authorization for use of their medical records in research (Figure 1). For the DELI cohort, we did not have information regarding previous history of bariatric surgery, endoscopic or surgical weight loss revisional procedures, or concomitant use of antiobesity medications.
		(*b*) *Cohort study*—For matched studies, give matching criteria and number of exposed and unexposed<br>*Case-control study*—For matched studies, give matching criteria and the number of controls per case	NA	
Variables	7	Clearly define all outcomes, exposures, predictors, potential confounders, and effect modifiers. Give diagnostic criteria, if applicable	9	The study's primary endpoint was the total body weight loss percentage (TBWL%) at 6 months of follow-up for patients participating in either the IPLI or the DELI program.

Data sources/ measurement	8*	For each variable of interest, give sources of data and details of methods of assessment (measurement). Describe comparability of assessment methods if there is more than one group	*9*
Bias	9	Describe any efforts to address potential sources of bias	9
Study size	10	Explain how the study size was arrived at	Figure 1

Continued on next page

Quantitative variables	11	Explain how quantitative variables were handled in the analyses. If applicable, describe which groupings were chosen and why	9	
Statistical methods	12	(*a*) Describe all statistical methods, including those used to control for confounding	9	
		(*b*) Describe any methods used to examine subgroups and interactions	9	
		(*c*) Explain how missing data were addressed	9	For the primary endpoint, we used 10 multiple imputation datasets for missing data using the mice package in R.
		(*d*) *Cohort study*—If applicable, explain how loss to follow-up was addressed<br>*Case-control study*—If applicable, explain how matching of cases and controls was addressed<br>*Cross-sectional study*—If applicable, describe analytical methods taking account of sampling strategy		
		(*e*) Describe any sensitivity analyses		
**Results**				
Participants	13*	(a) Report numbers of individuals at each stage of study—eg numbers potentially eligible, examined for eligibility, confirmed eligible, included in the study, completing follow-up, and analysed	10	Of the individuals selected at baseline from both cohorts, 93.1%% (47,524 /51,026) met the starting criteria in both the IPLI and DELI programs (Figure 1).
		(b) Give reasons for non-participation at each stage	10	
		(c) Consider use of a flow diagram	Figure 1	
Descriptive data	14*	(a) Give characteristics of study participants (eg demographic, clinical, social) and information on exposures and potential confounders	10	
		(b) Indicate number of participants with missing data for each variable of interest		
		(c) *Cohort study*—Summarise follow-up time (eg, average and total amount)		
Outcome data	15*	*Cohort study*—Report numbers of outcome events or summary measures over time	*10*	
		Case-control study—Report numbers in each exposure category, or summary measures of exposure		
		Cross-sectional study—Report numbers of outcome events or summary measures		
Main results	16	(*a*) Give unadjusted estimates and, if applicable, confounder-adjusted estimates and their precision (eg, 95% confidence interval). Make clear which confounders were adjusted for and why they were	10	

	included
	(*b*) Report category boundaries when continuous variables were categorized
	(*c*) If relevant, consider translating estimates of relative risk into absolute risk for a meaningful time period

Continued on next page

Other analyses	17	Report other analyses done—eg analyses of subgroups and interactions, and sensitivity analyses	
**Discussion**			
Key results	18	Summarise key results with reference to study objectives	11
Limitations	19	Discuss limitations of the study, taking into account sources of potential bias or imprecision. Discuss both direction and magnitude of any potential bias	13
Interpretation	20	Give a cautious overall interpretation of results considering objectives, limitations, multiplicity of analyses, results from similar studies, and other relevant evidence	13-14
Generalisability	21	Discuss the generalisability (external validity) of the study results	13
**Other information**			
Funding	22	Give the source of funding and the role of the funders for the present study and, if applicable, for the original study on which the present article is based	2

*Give information separately for cases and controls in case-control studies and, if applicable, for exposed and unexposed groups in cohort and cross-sectional studies.

Note: An Explanation and Elaboration article discusses each checklist item and gives methodological background and published examples of transparent reporting. The STROBE checklist is best used in conjunction with this article (freely available on the Web sites of PLoS Medicine at http://www.plosmedicine.org/, Annals of Internal Medicine at http://www.annals.org/, and Epidemiology at http://www.epidem.com/). Information on the STROBE Initiative is available at www.strobe-statement.org.
